# INADVERTENT TATTOOING OF ADJACENT LARGE BOWEL: A CASE REPORT AND REVIEW OF LITERATURE

**DOI:** 10.1590/S0102-67202014000200017

**Published:** 2014

**Authors:** Itai GHERSIN, Gideon SROKA, Bassel HAJ, Dana Shaylovsky GHERSIN, Ibrahim MATTER

**Affiliations:** 1 Rappaport Faculty of Medicine, Technion, Israel Institute of Technology; 2 Department of Surgery, Bnai Zion Medical Center, Haifa, Israel

## INTRODUCTION

Marking of colonic lesions which require surgical resection prior to surgery is of extreme importance, especially since laparoscopic approach is becoming increasingly common in colonic resections. Endoscopic tattooing of lesions, using dyes such as India ink, is recommended in such cases^1^, and is currently the most commonly used marking technique. This procedure was found to be both effective and safe in several studies.^2,3^

Several side effects and complications of India ink tattooing have been reported. Among them are localized leakages of ink into the peritoneal cavity, which were mostly asymptomatic^3^, and transmural injection of India ink into adjacent structures, such as small bowel^4^ and rectus muscle.^5^ However, we were not able to find any reports describing transmural injection of India ink into adjacent segments of large bowel, which prompted us to submit our case.

We present the case of a patient who underwent endoscopic tattooing of a colonic lesion prior to surgery. At laparotomy we noticed that the India ink was injected through the colon wall into an adjacent segment of large bowel, thus leading to inaccurate marking of the lesion.

## CASE REPORT

A 75 year old woman, with a history of hypothyroidism and essential hypertension, underwent a screening colonoscopy for the first time in her life. It is worth noting that the patient was asymptomatic. Colonoscopy revealed two polyps which were deemed endoscopically unresectable: one at the cecum and one at 40 cm from the anus. Both were biopsied, and a marking with India ink was made distal to the lesion at 40 cm in order to easily locate it at surgery. Both biopsies showed tubulovillous adenoma with areas of high grade dysplasia.

Further workup, including complete blood count, liver enzymes, CEA levels, chest x-ray and abdominal CT, was normal.

It was decided to proceed to surgery. We initially attempted to perform a laparoscopic resection, but due to severe intra-abdominal adhesions a conversion to open laparotomy was made. At laparotomy, a dark discoloration was seen at the distal transverse colon, about 100 cm from the anus, with no tattooing noted distally. It is worth noting that the transverse colon was in contiguity with the descending colon. Due to the discrepancy between the area of tattooing according to the colonoscopy report and the tattooed segment visualized at laparotomy, the possibility of inadvertent transmural injection was considered, and as a result we performed a complete dissection of the left colon and sigma, which enabled us to palpate the small lesion in the descending colon. A subtotal colectomy was performed in order to remove of both colonic lesions. Primary functional end to end anastomosis between the terminal ileum and the sigmoid colon was constructed.

During examination of the surgical specimen the cecal lesion was easily found, while the more distal lesion was seen at the descending colon. The India ink marking was in the transverse colon. It was apparent that the inaccurate marking was a result of a transmural injection of India ink through the wall of the descending colon into the transverse colon. The surgical margins of resection appeared to be free of tumor.

Postoperatively the patient made an uneventful recovery. Upon histologic examination of the surgical specimen the distal lesion turned out to be a moderately differentiated adenocarcinoma that invaded the submucosal layer, without involvement of the muscular layer (T1). The cecal lesion was a tubulovillous adenoma with areas of high grade dysplasia. The proximal, distal and radial margins of resection were free of tumor, as were all 13 examined lymph nodes (N0).

## DISCUSSION

Colonic lesions that require surgical excision may be difficult to localize at surgery, especially in the laparoscopic approach, since the surgeon cannot palpate small colonic lesions. Hence it is crucial to localize lesions prior to surgery.

India ink tattooing is often used to mark a colonic lesion during endoscopy. Multiple studies have shown this technique to be effective and safe^2,3^, with minimal complications and side effects^6,7^. Some reports, however, have described several possible side effects and complications of this procedure. These include the development of reactive lymph node swelling^8^, idiopathic inflammatory bowel disease^9^, an inflammatory pseudotumor showing granulomatous inflammation on biopsy^10^, and clinically silent localized peritonitis^8^ following India ink tattooing.

Park et al^3^ reported that localized leakages of ink into the peritoneal cavity were identified in 6 out of 63 patients who underwent pre-operative colonic lesion marking with India ink. Five of these patients were asymptomatic, while the sixth complained of mild chilling, without fever or abdominal pain.

Transmural injection of India ink through the colon wall into adjacent structures has also been reported. Bahadursingh et al^4^ described inadvertent injection into the small bowel wall, which simulated intestinal infarction at laparotomy. Alba et al^5^ described a case of injection through the colon wall into the rectus muscle, causing a rectus muscle abscess.

In our case report, the injection of India ink into an adjacent large bowel segment probably occurred due to the presence of significant adhesions between the transverse colon and the descending colon. The discrepancy between the area of tattooing according to the colonoscopy report and the tattooed segment visualized at laparotomy led us to suspect in a marking error, and as a result we performed a complete dissection of the left colon and sigma, so we were able to palpate the small lesion in the descending colon. The conversion from laparoscopic to open surgery made it easier for us to recognize the marking error, as it is harder to notice such errors during laparoscopy.

## Figures and Tables

**Figure 1:**
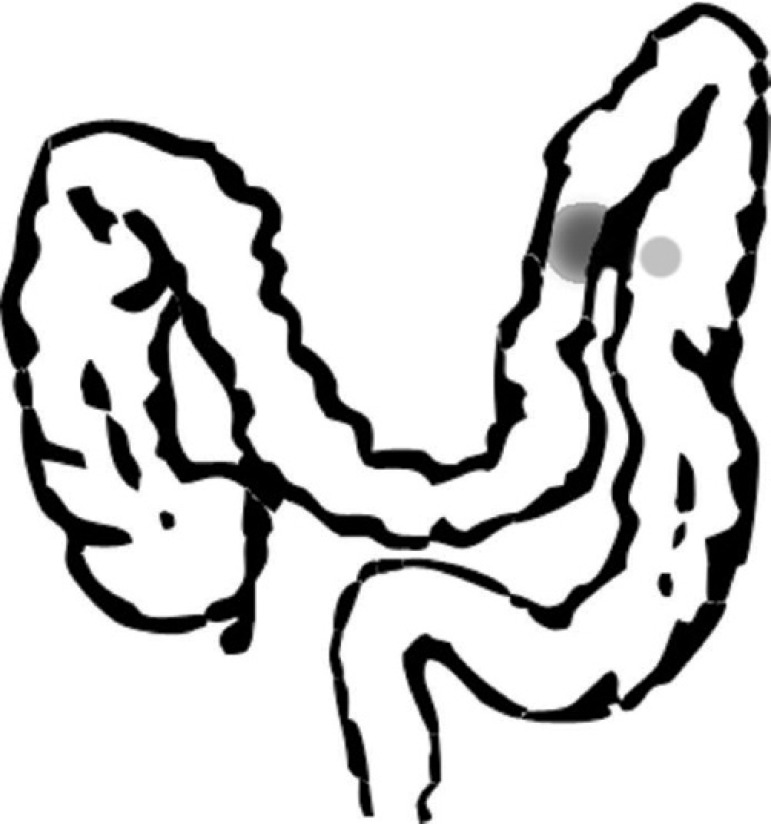
Inaccurate marking of a colonic lesion: this illustration demonstrates the location of India ink marking at the transverse colon (dark red) relative to the location of the lesion at the descending colon (light red). Illustration by Dana Shaylovsky Ghersin
